# Supplementary material for: Atomistic-Level Description of the Covalent Inhibition of SARS-CoV-2 Papain-like Protease
Source: Int J Mol Sci. 2022 May 23;23(10):5855. doi: 10.3390/ijms23105855 (PMC9143025; doi:10.3390/ijms23105855)
Supplement: Supplementary file 1 [file ijms-23-05855-s001.zip › ijms-1730062-supplementary.pdf]

Supporting Information for

Atomistic-level description of the covalent inhibition of SARS-CoV-2 papain-like protease

Cécilia Hognon [1], Marco Marazzi [1,2*] and Cristina García-Iriepa [1,2*]

[1] Universidad de Alcalá, Departamento de Química Analítica, Química Física e Ingeniería Química, Grupo de Reactividad y Estructura Molecular (RESMOL), Alcalá de Henares, Madrid, Spain; cecilia.hognon@uah.es

[2] Universidad de Alcalá, Instituto de Investigación Química ''Andrés M. del Río'' (IQAR), Alcalá de Henares, Madrid, Spain; marco.marazzi@uah.es; cristina.garciai@uah.es

* Correspondence: marco.marazzi@uah.es; cristina.garciai@uah.es

Content

1. RMSD analysis

2. MM-PBSA results

3. Time series data for the corresponding histograms

4. Ligand departure from the protease pocket in state D

5. Analysis of the thiol group conformations for Cys^{111}

6. QM/MM energy barrier for Cys^{111} deprotonation

7. Hydrogen-bond interaction analysis ligand-protease

8. BL2 loop conformation analysis for state D

9. Conformations of the ligand CH_2-CO-NH group

10. Distance analysis for state D between the Cys^{111}-S$^-$ and Vir251-C$_\beta$ or His^{272}-H$^+$

11. Hydrogen-bond interaction analysis between the vinyl methyl ester group and water molecules or protease side chains

12. Ligand force field details

1. RMSD simulation analysis

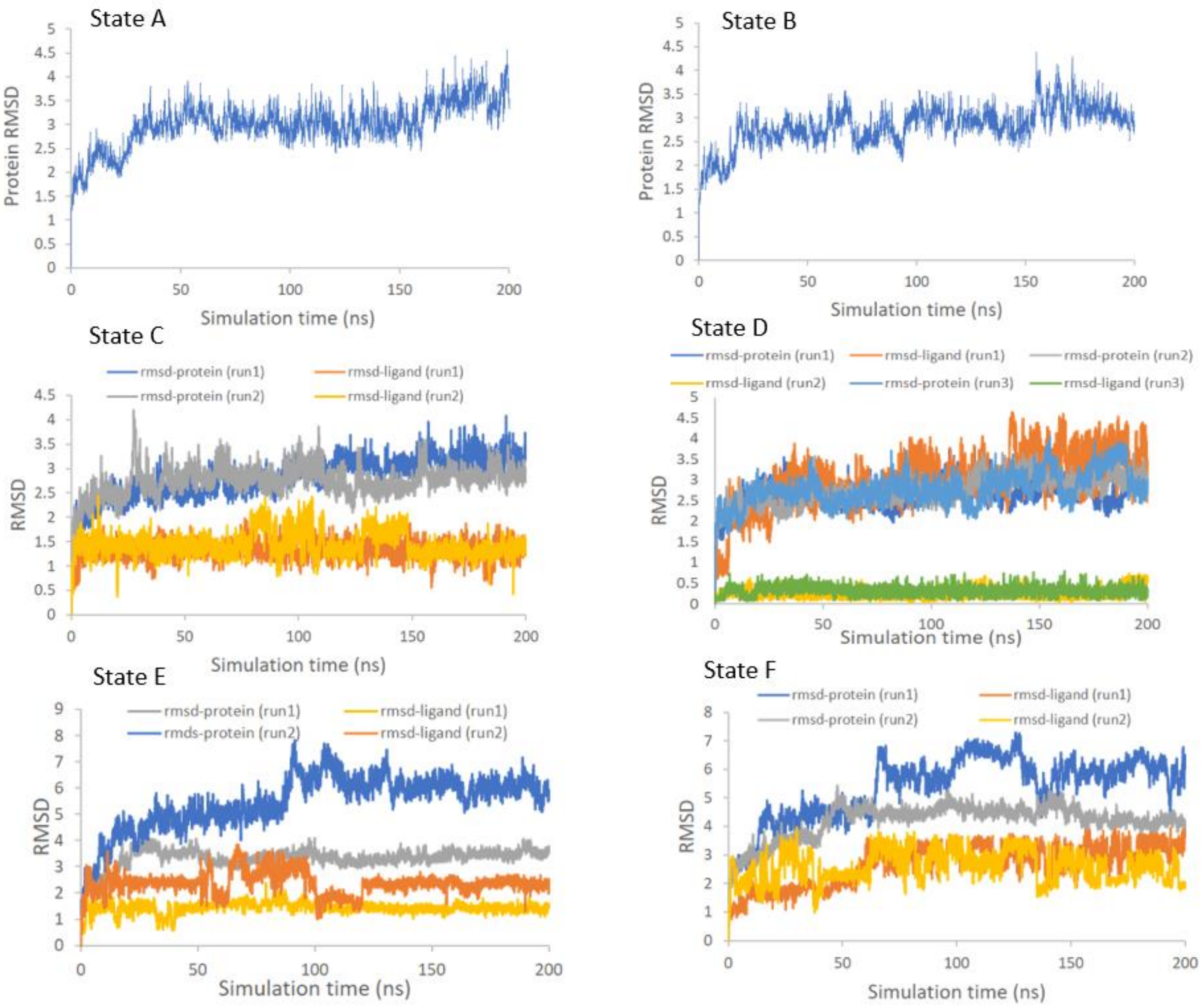

Figure S1. Protein RMSD analysis for each mechanism state under study (see Figure 1 for state nomenclature). For states C-F also the ligand RMSD analysis is depicted.

2. MMPBSA results

Table S1. MMPBSA results.

Step	Run	ΔG (kcal/mol)	TΔS (kcal/mol)	ΔH (kcal/mol)
A → C	1	-16.8722 ± 6.1415	-31.1789 ± 3.987	-48,0511
	2	-14.3902 ± 7.0834	-30.5637 ± 4.7092	-44,9539
B → D	2	5.6311 ± 8.9885	-28.7669 ± 5.1475	-23,1358
	3	-2.3268 ± 7.9644	-27.521 ± 4.2873	-29,8478

3. Time series data for the corresponding histograms

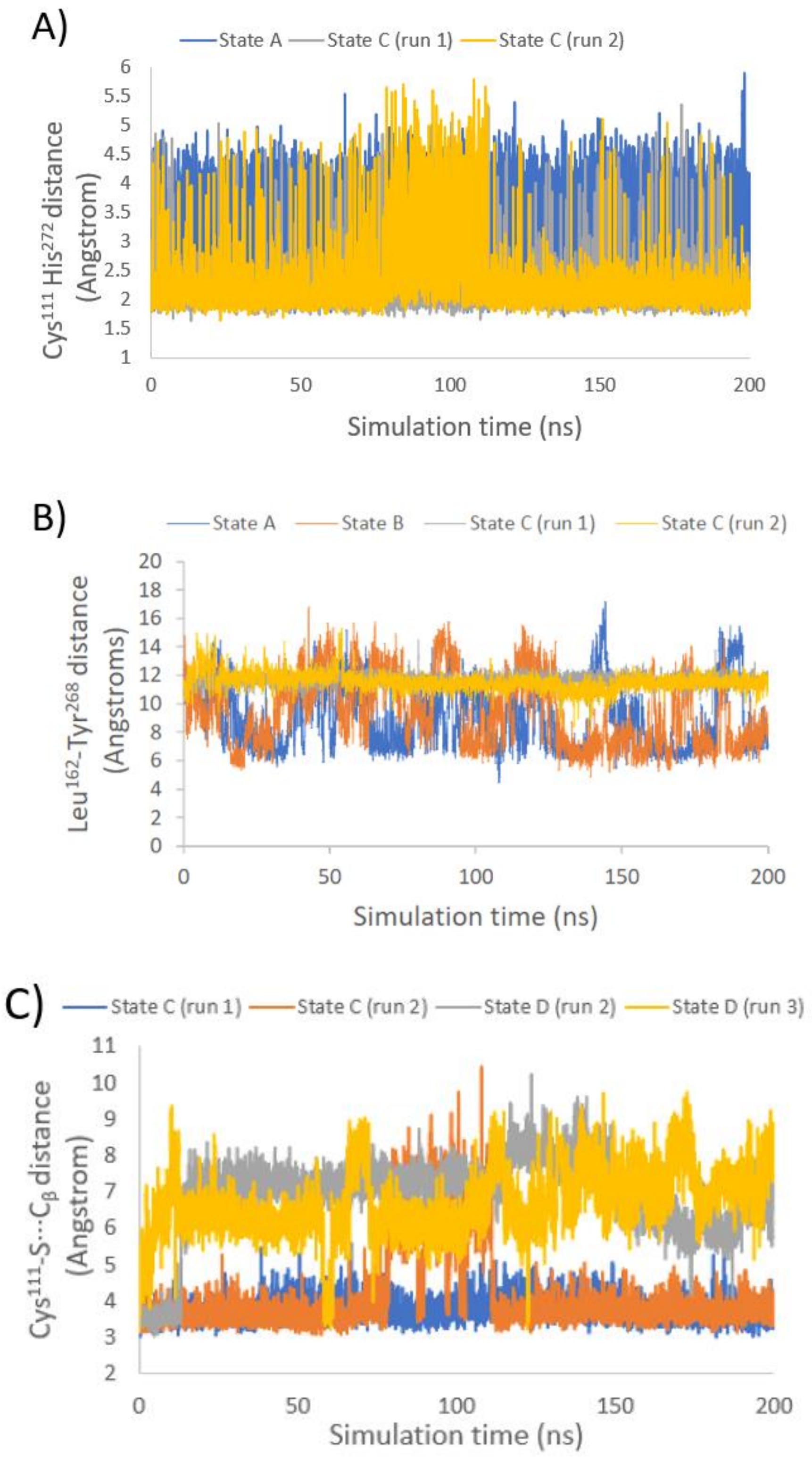

Figure S2. Time series data corresponding to histograms of A) figure 3B, B) figure 4C and C) figure 5B.

4. Ligand departure from the protease pocket in state D

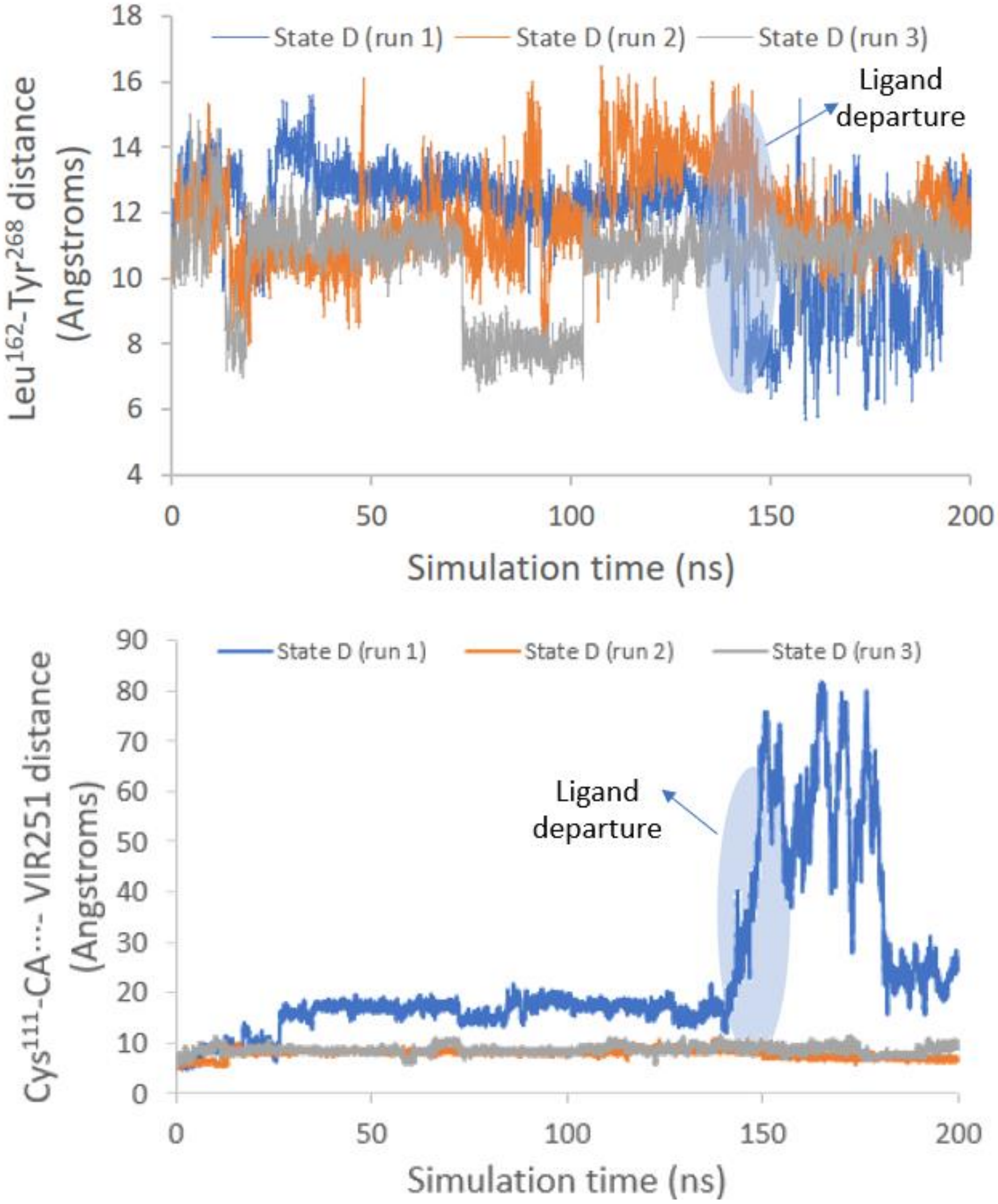

Figure S3. A) BL2 conformation measured through the Leu^{162}-Tyr^{268} distance and B) Cys^{111}-VIR251 distance along the simulation time, showing both an abrupt change of their values when the ligand moves out from the protease pocket.

5. Analysis of the thiol group conformations for Cys[111]

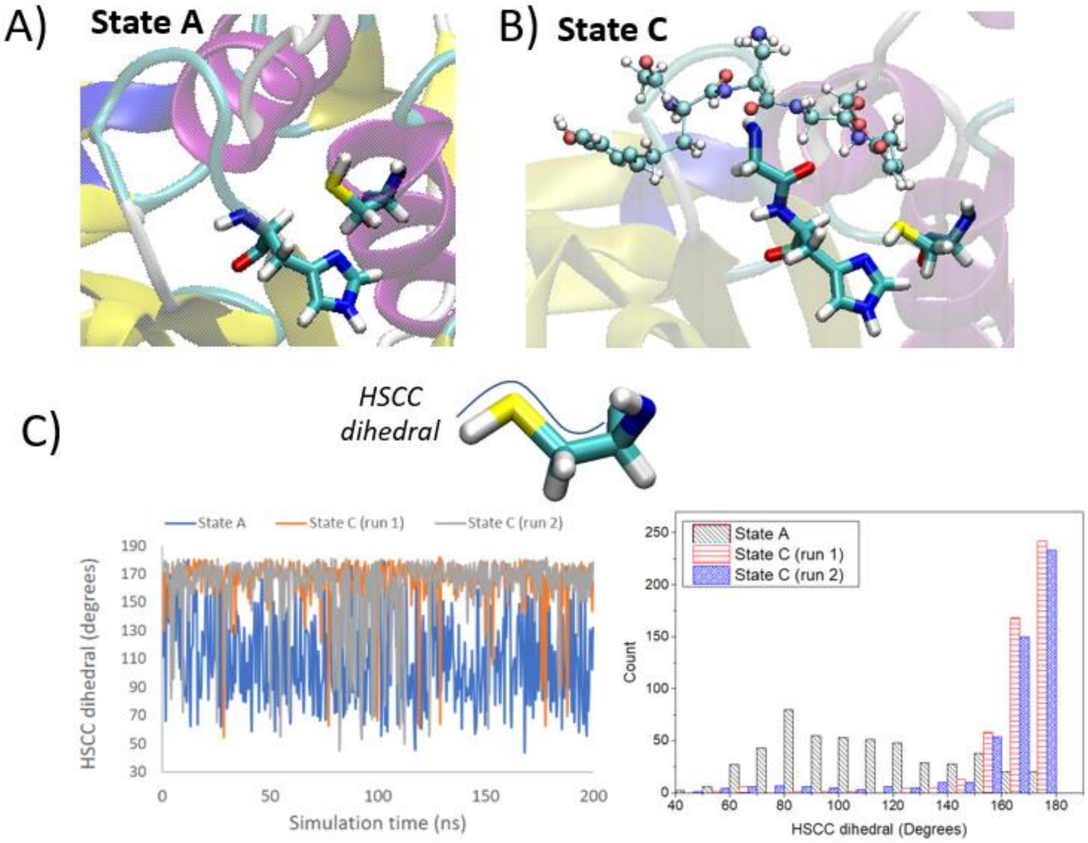

Figure S4. A) Representative snapshot of state A showing a particular conformation of Cys[111], pointing outwards His[272] and hence, hampering the hydrogen-bond interaction. B) Representative snapshot of state B, with the thiol group pointing towards the His[272]. Ligand is depicted in CPK representation showing its position on top of Cys[111] hampering it's the S-C free rotation. C) Time series and histogram of the HSCC dihedral angle (defined in the figure) showing the conformations populated during the MD simulations of states A and C.

6. QM/MM energy barrier for Cys[111] deprotonation

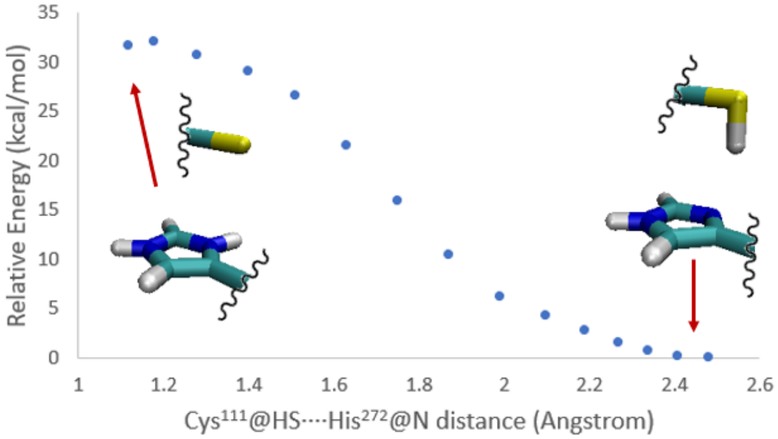

Figure S5. Energy barrier for the proton transfer process, from Cys[111] to His[272] computed for state A at the QM/MM level.

7. Hydrogen-bond interaction analysis

Table S2. Fraction of hydrogen-bond interactions found along the simulation time of each MD trajectory between the protease residues and the ligand.

Residue	State C (Run 1)	State C (Run 2)	State D (Run 2)	State D (Run 3)	State E (Run 1)	State E (Run 2)	State F (Run 1)	State F (Run 2)
Tyr 264	0.9158	0.6856	0.3016	0.6876	0.8654		0.8650	0.3174
Gly 271	0.7694	0.7776	0.1580	0.1990	0.7966	0.6394	0.6746	0.6798
Gly 163@O	0.6020	0.6344	0.5936	0.5630	0.7826	0.8106	0.7754	0.7598
Gly 163@H	0.3310	0.3216	0.5048	0.1560	0.5558	0.4406	0.4582	0.5256
Tyr 268	0.5048	0.4120	0.1358	0.3190	0.4722		0.1278	0.1598
Arg 166	0.3304	0.3256	<0.100	0.1504	0.4300	0.3868		
Tyr 273						0.6078		

8. BL2 loop conformation analysis for state D

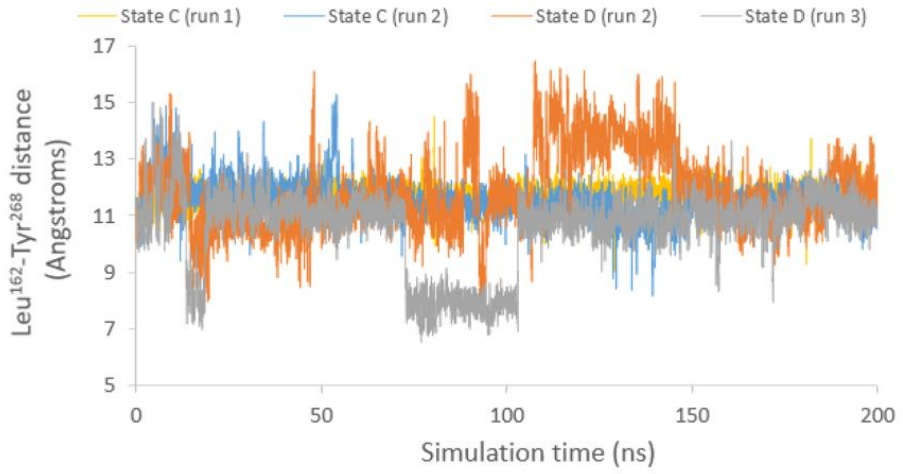

Figure S6. Leu^{162}-Tyr^{268} distance along the MD simulations of states C and D, showing a less stable conformation of the BL2 loop for state D.

9. Conformations of the ligand CH_2-CO-NH group

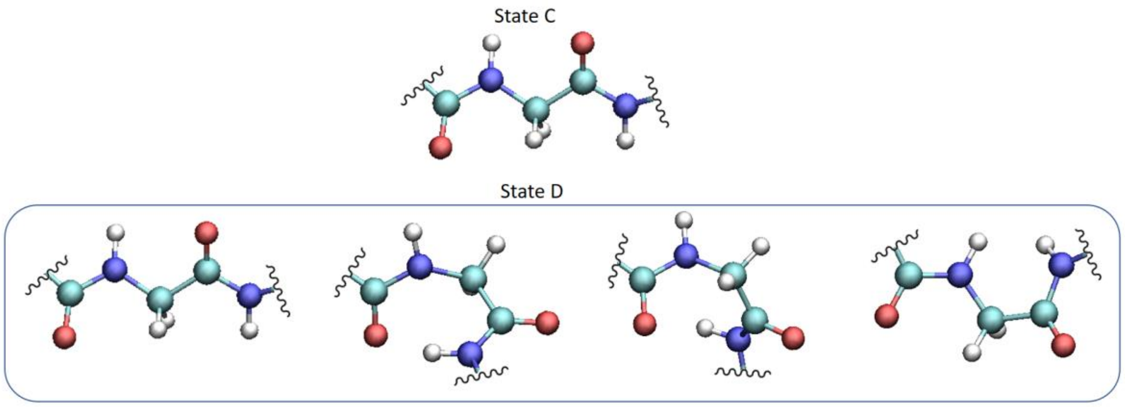

Figure S7. Schematic representations of the most populated conformation for the CH_2-CO-NH group of the ligand for states C and D.

10. Distance analysis for state D between the Cys^111^@S^-^ and Vir251@C$_β$ or His^272^@H^+^

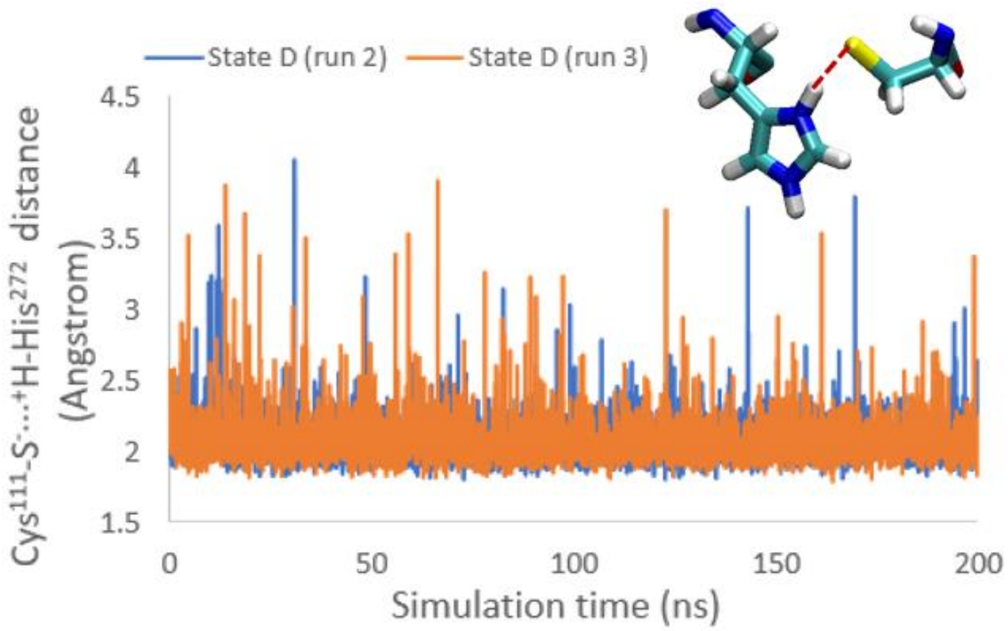

Figure S8. Distance between the negatively charged S atom of Cys^{111} and the transferred proton to His^{272} along the simulation time for the two independent simulations of state D.

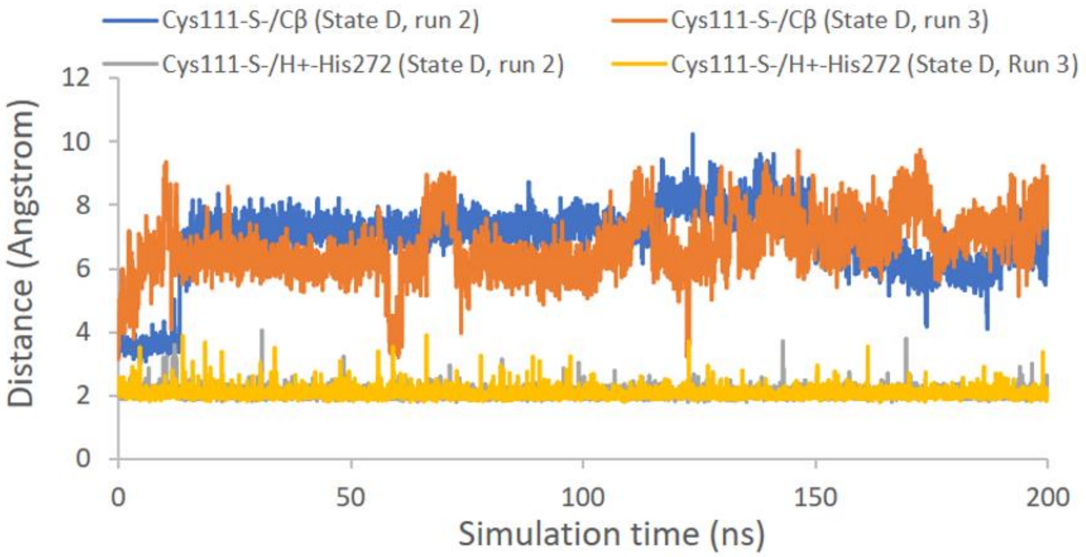

Figure S9. Cys^{111}-S^{-}···C$_β$-VIR251 and Cys^{111}-S^{-}···$^{+}$H-His^{272} distance along the MD simulations of state D.

11. Hydrogen-bond interaction analysis between the vinyl methyl ester group and water molecules or protease side chains

Table S3. Fraction of hydrogen-bond interactions between the oxygen of the carbonyl group or oxygen of the methoxy group conforming the vinyl methyl ester group with water molecules or protease residues along the MD simulation.

Residue	State C (Run 1)	State C (Run 2)	State D (Run 2)	State D (Run 3)	State E (Run 1)	State E (Run 2)
Carbonyl oxygen (C=O)	Water (1.2170)	Water (1.2206)	Water (0.7406) GLY^{160}@H (0.3836)	Water (1.1598)	Water (1.8496)	Water (1.7736)
Methoxy oxygen (OMe)					Water (0.4502)	Water (0.5310)

12. Ligand force field details

Table S4. Atom type, xyz coordinates of the optimized geometry and RESP charges for VIR251 state C and D.

Name	X	Y	Z	Atom type	Charge
**C1**	-6.4700	-1.9210	-0.2790	c	0.880559
**O1**	-6.6230	-1.0080	-1.1040	o	-0.664432
**C2**	-7.4510	-3.0730	-0.1840	c3	-0.486499
**N1**	-5.4270	-1.9560	0.5900	n	-0.809988
**C3**	-4.3860	-0.9340	0.6550	c3	0.242707
**C4**	-3.0300	-1.6600	0.6170	c	0.483729
**C5**	-4.5310	-0.0850	1.9360	c3	0.008453
**C6**	-3.5510	1.1020	2.0470	c3	-0.132125
**C7**	-3.7470	2.1890	1.0060	ca	0.058878
**C8**	-2.7860	2.4390	0.0140	ca	-0.178927
**C9**	-2.9620	3.4440	-0.9400	ca	-0.331208
**C10**	-4.1190	4.2270	-0.9150	ca	0.430254
**C11**	-5.0930	4.0010	0.0650	ca	-0.331208
**C12**	-4.8990	2.9920	1.0110	ca	-0.178927
**O2**	-4.2460	5.2060	-1.8720	oh	-0.572924
**O3**	-2.7470	-2.4990	1.4830	o	-0.587626
**N2**	-2.2090	-1.3220	-0.4010	n	-0.396462
**C13**	-0.8790	-1.8790	-0.6020	c3	-0.012751
**C14**	0.1140	-0.7160	-0.7440	c	0.450022
**O4**	-0.1600	0.2640	-1.4520	o	-0.544209
**C15**	-0.8020	-2.7680	-1.8650	c3	0.256483
**N3**	-1.6720	-3.9400	-1.7430	n3	-0.967320

**N4**	1.2840	-0.8470	-0.0920	n	-0.288379
**C16**	2.3360	0.1450	-0.1740	c3	-0.329888
**C17**	3.4940	-0.2830	0.7300	c	0.752807
**O5**	3.4430	-1.3280	1.3890	o	-0.554363
**N5**	4.5440	0.5670	0.7460	n	-0.763331
**C18**	5.7520	0.3510	1.5410	c3	0.316863
**C19**	6.9170	-0.0610	0.6840	c2	-0.003945
**C20**	8.0930	0.5810	0.6580	ce	-0.588760
**C21**	9.2090	0.1160	-0.1960	c	0.977035
**O6**	10.2760	0.9340	-0.0790	os	-0.437097
**O7**	9.1970	-0.8690	-0.9200	o	-0.626795
**C22**	11.4410	0.5960	-0.8630	c3	-0.039803
**H1**	4.5100	1.3910	0.1590	hn	0.385284
**H2**	1.4720	-1.6420	0.5100	hn	0.223695
**H3**	-1.2620	-4.6250	-1.1120	hn	0.368908
**H4**	-1.7640	-4.3970	-2.6460	hn	0.368908
**H5**	-5.3510	-2.7280	1.2390	hn	0.394975
**H6**	-2.5050	-0.6120	-1.0590	hn	0.296740
**H7**	-5.0800	5.6830	-1.7520	ho	0.386050
**H8**	-8.4410	-2.6720	0.0520	hc	0.131871
**H9**	-7.1780	-3.8140	0.5710	hc	0.131871
**H10**	-7.5140	-3.5630	-1.1590	hc	0.131871
**H11**	-1.8780	1.8420	-0.0200	ha	0.162798
**H12**	-2.2090	3.6270	-1.7010	ha	0.187063
**H13**	-5.9920	4.6110	0.0920	ha	0.187063
**H14**	-5.6620	2.8360	1.7700	ha	0.162798
**H15**	0.2560	-3.0220	-2.0320	h1	0.018087
**H16**	-1.1340	-2.1780	-2.7250	h1	0.018087
**H17**	1.9650	1.1270	0.1400	h1	0.164031
**H18**	2.6930	0.2490	-1.2060	h1	0.164031
**H19**	8.2730	1.4670	1.2600	ha	0.207645
**H20**	12.1650	1.3840	-0.6580	h1	0.092731
**H21**	11.1890	0.5720	-1.9260	h1	0.092731
**H22**	11.8330	-0.3760	-0.5550	h1	0.092731
**H23**	-4.5140	-0.3030	-0.2270	h1	0.094168
**H24**	-4.3980	-0.7440	2.8020	hc	0.014723
**H25**	-5.5630	0.2820	1.9660	hc	0.014723
**H26**	-2.5180	0.7360	2.0030	hc	0.055631
**H27**	-3.6760	1.5330	3.0480	hc	0.055631
**H28**	5.5200	-0.4420	2.2580	h1	0.035621
**H29**	5.9800	1.2630	2.1010	h1	0.035621
**H30**	6.7740	-0.9390	0.0560	ha	0.135263
**H31**	-0.6490	-2.4800	0.2820	h1	0.157827

Table S5. Atom type, xyz coordinates of the optimized geometry and RESP charges for VIR251 state E.

Name	X	Y	Z	Atom type	Charge
**N1**	-6.9380	-1.5930	0.2770	N	-0.395868
**C1**	-6.9770	-2.0750	-1.1140	CT	-0.047432
**H1**	-8.0070	-2.3520	-1.3950	H1	0.081191
**C2**	-6.5640	-0.9100	-2.0680	CT	-0.152196
**S1**	-4.9380	-0.0640	-1.7480	S	-0.405786
**C3**	-6.1540	-3.3760	-1.3530	C	0.625516
**O1**	-6.7290	-4.4140	-1.7390	O	-0.632980
**C4**	7.6370	-1.4410	1.6000	C	0.772756
**O2**	8.0690	-0.3100	1.4160	O	-0.628271
**C5**	8.4760	-2.5280	2.2510	CT	-0.498254
**N2**	6.3670	-1.7980	1.2760	N	-0.579361
**C6**	5.3630	-0.9010	0.6600	CT	0.200970
**C7**	4.2310	-0.6150	1.6470	C	0.525652
**C8**	4.7720	-1.4790	-0.6630	CT	0.000758
**C9**	5.8440	-1.8940	-1.7120	CT	-0.104643
**C10**	6.8100	-0.7740	-2.1080	CA	0.104245
**C11**	6.3340	0.3900	-2.7370	CA	-0.237827
**C12**	7.2270	1.4010	-3.1220	CA	-0.292525
**C13**	8.6010	1.2550	-2.8850	CA	0.422208
**C14**	9.0810	0.0940	-2.2600	CA	-0.292525
**C15**	8.1880	-0.9150	-1.8760	CA	-0.237827
**O3**	9.4690	2.2400	-3.2730	OH	-0.582249
**O4**	3.9100	-1.4000	2.5330	O	-0.571752
**N3**	3.5220	0.5170	1.4000	N	-0.608273
**C16**	2.4350	1.0380	2.2560	CT	-0.013109
**C17**	1.2770	1.5200	1.3870	C	0.669608
**O5**	1.1720	2.6800	1.0010	O	-0.611548
**C18**	2.9280	2.1630	3.2060	CT	0.276963
**N4**	3.9500	1.6640	4.1590	NT	-0.944886
**N5**	0.4550	0.5360	0.9360	N	-0.631801
**C19**	-0.6750	0.7370	0.0050	CT	-0.057608
**C20**	-2.0280	0.5510	0.6810	C	0.705872
**O6**	-2.2540	-0.3930	1.4270	O	-0.633145
**N6**	-2.9980	1.4330	0.3040	N	-0.534075
**C21**	-4.4080	1.2660	0.6740	CT	-0.009154
**C22**	-5.3560	1.3530	-0.5580	CT	0.561084
**C23**	-5.3350	2.7100	-1.2220	CM	-1.169581
**C24**	-5.9700	3.7570	-0.5540	C	0.964388
**O7**	-5.7180	5.0400	-1.1140	OS	-0.477979
**O8**	-6.7170	3.6860	0.4760	O	-0.789536
**C25**	-6.2080	6.1630	-0.3660	CT	-0.005846
**H2**	-2.7670	2.2250	-0.2940	H	0.323893

**H3**	0.6210	-0.4070	1.2600	H	0.348382
**H4**	3.5620	0.9270	4.7550	H	0.357922
**H5**	4.2710	2.4180	4.7790	H	0.357922
**H6**	6.0670	-2.7400	1.4850	H	0.316155
**H7**	3.8100	1.1110	0.6250	H	0.369668
**H8**	10.3820	1.9940	-3.0410	HO	0.389405
**H9**	9.4240	-2.6470	1.6980	HC	0.134055
**H10**	7.9600	-3.4970	2.2370	HC	0.134055
**H11**	8.7000	-2.2650	3.2810	HC	0.134055
**H12**	5.2650	0.5200	-2.9390	HA	0.186364
**H13**	6.8530	2.3100	-3.6120	HA	0.179764
**H14**	10.1560	-0.0190	-2.0830	HA	0.179764
**H15**	8.5770	-1.8150	-1.4010	HA	0.186364
**H16**	2.0880	2.5630	3.7790	H1	0.014315
**H17**	3.3750	2.9800	2.6150	H1	0.014315
**H18**	-0.6130	0.0020	-0.7960	H1	0.108304
**H19**	-0.6340	1.7470	-0.4340	H1	0.108304
**H20**	-5.6790	7.0430	-0.7550	H1	0.054318
**H21**	-6.0010	6.0520	0.7100	H1	0.054318
**H22**	-7.2940	6.3000	-0.4990	H1	0.054318
**H23**	5.8400	0.0680	0.4310	H1	0.091267
**H24**	4.1020	-0.7280	-1.1140	HC	0.020257
**H25**	4.1560	-2.3530	-0.4320	HC	0.020257
**H26**	5.3250	-2.2410	-2.6220	HC	0.033246
**H27**	6.4070	-2.7530	-1.3370	HC	0.033246
**H28**	-4.7120	2.0720	1.3620	H1	0.064090
**H29**	-4.4900	0.3080	1.2040	H1	0.064090
**H30**	2.0430	0.2180	2.8740	H1	0.138059
**H31**	-7.3560	-0.1470	-2.0810	H1	0.132671
**H32**	-6.4830	-1.3140	-3.0880	H1	0.132671
**H33**	-6.3720	1.1630	-0.1710	H1	0.060992
**H38**	-6.6460	-0.6280	0.4050	H	0.207071
**H42**	-4.6500	2.9090	-2.0470	HA	0.230939

Table S6. Atom type, xyz coordinates of the optimized geometry and RESP charges for VIR251 state F.

Name	X	Y	Z	Atom type	Charge
**N1**	-6.9060	-1.6420	0.3390	N	-0.532183
**C1**	-6.9230	-2.1450	-1.0480	CT	0.051602
**H1**	-7.9450	-2.4440	-1.3360	H1	0.085178
**C2**	-6.5250	-0.9900	-2.0210	CT	-0.168901
**S1**	-4.8990	-0.1350	-1.7140	S	-0.280978
**C3**	-6.0690	-3.4280	-1.2700	C	0.588075
**O1**	-6.6190	-4.4730	-1.6810	O	-0.605077

**C4**	7.6270	-1.4040	1.6520	C	0.783638
**O2**	8.0660	-0.2850	1.4380	O	-0.619624
**C5**	8.4570	-2.4830	2.3350	CT	-0.536694
**N2**	6.3540	-1.7660	1.3280	N	-0.573136
**C6**	5.3600	-0.8790	0.6840	CT	0.188440
**C7**	4.2240	-0.5590	1.6580	C	0.528873
**C8**	4.7730	-1.4870	-0.6260	CT	-0.002599
**C9**	5.8490	-1.9360	-1.6610	CT	-0.103672
**C10**	6.8240	-0.8310	-2.0800	CA	0.101005
**C11**	6.3590	0.3200	-2.7420	CA	-0.234686
**C12**	7.2600	1.3160	-3.1470	CA	-0.296047
**C13**	8.6330	1.1660	-2.8990	CA	0.435855
**C14**	9.1030	0.0190	-2.2420	CA	-0.296047
**C15**	8.1980	-0.9750	-1.8310	CA	-0.234686
**O3**	9.5120	2.1410	-3.3000	OH	-0.590521
**O4**	3.8950	-1.3200	2.5660	O	-0.565310
**N3**	3.5210	0.5730	1.3750	N	-0.598755
**C16**	2.4340	1.1190	2.2150	CT	-0.022317
**C17**	1.2800	1.5870	1.3270	C	0.665286
**O5**	1.1800	2.7400	0.9140	O	-0.608917
**C18**	2.9330	2.2680	3.1400	CT	0.283176
**N4**	3.9440	1.7850	4.1120	NT	-0.941680
**N5**	0.4560	0.5980	0.8960	N	-0.596828
**C19**	-0.6670	0.7810	-0.0460	CT	-0.057821
**C20**	-2.0260	0.6200	0.6270	C	0.688906
**O6**	-2.2630	-0.3010	1.3970	O	-0.586121
**N6**	-2.9780	1.5120	0.1970	N	-0.542234
**C21**	-4.3910	1.4290	0.5780	CT	0.019361
**C22**	-5.3660	1.3220	-0.6280	CT	0.075540
**C23**	-5.4760	2.6230	-1.4640	CT	-0.611921
**C24**	-6.1200	3.7510	-0.6550	C	0.980876
**O7**	-5.5070	4.9380	-0.8570	OS	-0.451921
**O8**	-7.0880	3.6030	0.0800	O	-0.622342
**C25**	-6.0610	6.0850	-0.1460	CT	-0.041163
**H2**	-2.6740	2.3090	-0.3580	H	0.334897
**H3**	0.6210	-0.3480	1.2440	H	0.346424
**H4**	3.5490	1.0670	4.7210	H	0.364190
**H5**	4.2640	2.5560	4.7120	H	0.364190
**H6**	6.0520	-2.6990	1.5640	H	0.318191
**H7**	3.8280	1.1430	0.5930	H	0.374131
**H8**	10.4320	1.9030	-3.0550	HO	0.400920
**H9**	9.4160	-2.6130	1.7930	HC	0.148401
**H10**	7.9390	-3.4500	2.3440	HC	0.148401
**H11**	8.6820	-2.1880	3.3720	HC	0.148401
**H12**	5.3010	0.4460	-2.9510	HA	0.187440

**H13**	6.8980	2.2130	-3.6630	HA	0.183363
**H14**	10.1800	-0.1070	-2.0510	HA	0.183363
**H15**	8.5920	-1.8620	-1.3360	HA	0.187440
**H16**	2.0860	2.6840	3.6950	H1	0.012748
**H17**	3.3860	3.0640	2.5270	H1	0.012748
**H18**	-0.5990	0.0120	-0.8310	H1	0.110117
**H19**	-0.6110	1.7730	-0.5130	H1	0.110117
**H20**	-5.4070	6.9250	-0.4010	H1	0.100978
**H21**	-6.0490	5.9000	0.9380	H1	0.100978
**H22**	-7.0890	6.2790	-0.4800	H1	0.100978
**H23**	5.8500	0.0820	0.4350	H1	0.091801
**H24**	4.1180	-0.7470	-1.1000	HC	0.015572
**H25**	4.1600	-2.3550	-0.3740	HC	0.015572
**H26**	5.3440	-2.3060	-2.5600	HC	0.040874
**H27**	6.4160	-2.7900	-1.2540	HC	0.040874
**H28**	-4.6690	2.3120	1.1810	H1	0.092366
**H29**	-4.4940	0.5460	1.2220	H1	0.092366
**H30**	2.0370	0.3150	2.8510	H1	0.137176
**H31**	-7.3220	-0.2330	-2.0420	H1	0.142467
**H32**	-6.4440	-1.4050	-3.0350	H1	0.142467
**H33**	-6.3680	1.1310	-0.2140	H1	0.133942
**H34**	-6.1340	2.4420	-2.3300	HC	0.186709
**H35**	-4.5040	2.9450	-1.8630	HC	0.186709
**H40**	-6.7630	-0.6430	0.4530	H	0.289036
